# Characterization of Physicochemical and Sensory Properties of Cheeses Added with Bovine Colostrum

**DOI:** 10.3390/foods12244474

**Published:** 2023-12-14

**Authors:** Idiana de Macêdo Barbosa, Katya Anaya, Cláudia Souza Macêdo, Robson Rogério Pessoa Coelho, Claudio Cipolat-Gotet, Emerson Gabriel dos Santos Oliveira Silva, Nkarthe Guerra Araújo, Bruna Maria Emerenciano das Chagas, Juliana Paula Felipe de Oliveira, Cleube Andrade Boari, Danielle Cavalcanti Sales, Emmanuella de Oliveira Moura Araújo, Josemir Araújo Neves, Adriano Henrique do Nascimento Rangel

**Affiliations:** 1 Academic Unit Specialized in Agricultural, Federal University of Rio Grande do Norte (UFRN), Macaíba 59280-000, RN, Brazil; idiana_corrego@yahoo.com.br (I.d.M.B.); adrianohrangel@yahoo.com.br (A.H.d.N.R.); 2 Health Sciences College of Trairi, Federal University of Rio Grande do Norte, Santa Cruz 59200-000, RN, Brazil; katya.anaya@ufrn.br; 3 Department of Veterinary Science, University of Parma, 43121 Parma, Italy; 4 Infrastructure Superintendence, Federal University of Rio Grande do Norte, Natal 59078-970, RN, Brazil; 5 Rural Health and Technology Center, Federal University of Campina Grande, Patos 58708-110, PB, Brazil; 6 Department of Animal Science, Federal University of the Jequitinhonha and Mucuri Valleys, Diamantina 39100-000, MG, Brazil; 7 Agricultural Research Company of Rio Grande do Norte, Natal 59062-500, RN, Brazil

**Keywords:** sensory evaluation, dairy product, cheese maturation, food composition

## Abstract

The objective of this study was to develop fresh and matured cheeses with different bovine colostrum levels, aiming to promote the consumption of dairy products with the addition of colostrum. Four different cheese formulations were produced with a mixture of 0:100, 15:85, 20:80, and 25:75, bovine colostrum:milk (*v*:*v*), and aged for 0, 10, 20, and 40 days. Milk, colostrum, and fresh and matured cheeses were submitted to physicochemical characterization. Moreover, microbiological quality, yield, texture profile, color, and sensory acceptance of cheese samples were evaluated. Colostrum supplementation favored low acidity, high moisture, a pH range of 5.0–6.2, and water activity of 0.94–99. Sensory attributes and overall evaluation of all cheese formulations achieved an Acceptability Index above 70, indicating good acceptability. Since cheese with colostrum presented the potential to be used as human food, assessing the presence of colostrum bioactive components in those dairy products is a promising goal for further research.

## 1. Introduction

The colostrum is the secretion produced by the mammary gland immediately after birth [1]. It represents a rich source of vitamin A with relatively high concentrations of caseins and albumin, containing many essential nutrients in higher concentrations than those usually found in mature milk [1,2]. For instance, Jersey cows’ colostrum 24 h after birth presents 4% fat, 15% proteins (12% caseins), 1,5% lactose and 489 IU/dL vitamin A [3]. Compared to milk, colostrum generally contains less lactose and has a higher content of other components such as fat, protein, ash, vitamins, hormones, and immunoglobulins. After three days, the lactose content increases, while the percentage of other components gradually decreases [1,4]. In addition, bovine colostrum (BC) contains bioactive components in relevant amounts, including growth factors, immunoglobulins, lactoperoxidase, lysozyme, lactoferrin, nucleosides, vitamins, peptides, and oligosaccharides, which are extremely relevant to health [5]. Immunoglobulins can account for more than 80% of total proteins in BC; therefore, its commercial value is currently quoted by the protein concentration and immunoglobulin levels, especially IgG [6,7]. Recent research has shown that immune defense proteins in bovine colostrum may be related to protective effects against respiratory diseases such as those caused by SARS-CoV-2 infection (COVID-19) [8]. Bioactive molecules are allegedly responsible for the effect of passive immunity when BC is consumed by humans [9]. Despite considerable differences between BC and human colostrum compositions, BC has good tolerability in the human organism [6] and it is well accepted by most consumers [7].

The demand for innovative foods grows every day as consumers consider nutrition to address a wide range of health conditions. In this way, industries and research centers promote initiatives to support the production of nutraceuticals and functional foods. Food companies are developing their product lines with a primary focus on promoting people’s health [10]. In this sense, some products based on bovine colostrum have been developed and are already commercialized as food supplements in countries such as New Zealand, United States, Europe, and China [11]. Nevertheless, a wide variation in the bioactivity was detected in commercially available colostrum products such as powders and capsules [7]. A crescent number of patents has been registered across the globe claiming health benefits to colostrum products, with emerging processing technologies being developed to preserve the therapeutic properties of BC bioactive compounds [12].

Regarding food products, yogurts [13,14], mixtures for beverages [15], cheeses [16,17,18], ice cream [19], nutritional bars, jellies, and ready-to-drink beverages have used or might use BC as a functional ingredient [20]. Dairy products, such as cheeses, are a valuable source of proteins, lipids, vitamins, and minerals, and their global consumption is expected to increase by around 13.8% between 2019 and 2029 [21]. Thus, the production of mixed cheese with milk and colostrum has emerged as an alternative way of utilizing surplus colostrum and an innovation in the dairy industry for providing possible benefits because of the presence of immunoglobulins, which adhere to the intestinal mucosa, functioning as a protective layer and preventing pathogenic microorganisms from colonizing [22].

Considering the worldwide consumption of cheese, developing this type of product with the inclusion of colostrum could be an innovative alternative to utilizing the surplus production of this raw material. Furthermore, once the health benefits of colostrum and its potential for new products are understood, there is an opportunity for industries to cater to different consumer profiles [18].

In this sense, this study aimed at (i) developing fresh and mature cheeses added with different bovine colostrum levels and (ii) characterizing the physical-chemical and sensory properties of these cheeses.

## 2. Materials and Methods

### 2.1. Milk and Colostrum Sampling and Thermal Processing

The milk and colostrum were collected on a commercial farm located in the municipality of São Gonçalo do Amarante, Rio Grande do Norte, Brazil, between January and November 2019. The herd consisted of 62 Jersey cows (30 primiparous and 32 multiparous), managed under a Compost Barn system. Colostrum samples were collected at second and third milking after calving (up to 24 h) in plastic bottles with a capacity of one liter, labeled and packed in isothermal boxes at a temperature of 4 to 5 °C, transported to the Milk Quality Laboratory (LABOLEITE) of the Federal University of Rio Grande do Norte at the Macaíba Campus, and then stored at −18 °C. The colostrum was subsequently thawed in a cold chamber at a refrigeration temperature of 5 °C to perform thermal processing, physical-chemical analysis, and cheese-making. Subsequently, milk and colostrum mixtures in the proportions 100:0, 85:15, 80:20, and 75:25 (*v*:*v*; milk:colostrum, respectively) were subjected to heat treatment (60 °C for 45 min) according to Das and Seth [23], aiming at ensuring the microbial quality of the raw material to be used in cheese-making, while preserving the IgG and IgA immunoglobulins, with no visual protein coagulation.

The preliminary pasteurization tests showed high thermal instability of the second milking colostrum, which led us to follow the cheese production stage with only the third milking colostrum.

### 2.2. Physical-Chemical Analysis of Milk and Colostrum

Milk and colostrum were analyzed for fat, total protein, lactose, casein, total solids, and milk solids non-fat using an infrared spectrophotometer (Dairy Spect^®^, Bentley Instruments Inc., Chaska, MI, USA). The pH of milk and colostrum samples was measured using a digital pH meter (Lucadema, São José do Rio Preto-SP, Brazil). Acidity was determined according to the standards of the Instituto Adolfo Lutz [24] using a Dornic Acidimeter (CAP-LAB, Ipiranga-SP, Brazil), and the result was expressed in g of lactic acid/100 mL. All these measurements were performed in triplicate.

### 2.3. Experimental Treatments and Cheese-Making

This study tested four treatments characterized by a different colostrum addition (0, 15, 20, 25 mL 100 mL^−1^, respectively named 0, 1, 2, 3). Cheese produced using 100 mL of milk was defined as the control treatment. The colostrum proportions were chosen according to preliminary tests on the thermal stability of the mixture of colostrum and milk. Increasing proportions of colostrum promoted the coagulation of the mixture during the pasteurization. Furthermore, higher amounts of colostrum could potentially lead to remarkable sensory changes, negatively influencing the final acceptance of the products.

Different ripening periods were assessed for each treatment, from 0 days (fresh; F) to 10, 20, and 40 days, respectively named A, B, and C. For each treatment, cheese-making was performed following several procedures: after pasteurization, milk with or without colostrum was cooled to 37 °C, inoculated with a mixed starter culture (*Lactoccocus lactis subsp. Lactococcus lactis subsp. cremoris, Streptococcus salivarius subsp. thermophilus*) (Vilac Foods^®^, Macaíba, Brazil), in the proportion of 0.6 mL/10 L of milk, then allowed to rest for 30 min. Following the manufacturer’s instructions, commercial 0.04 g 100 g^−1^ calcium chloride and 0.01 g 100 g^−1^ calf rennet (LacRen 1000 IMCU/mL; Vilac Foods^®^, Brazil) were homogenized and added to the vat, and milk was left for coagulation (40 to 60 min). The final curd pH was monitored to be no lower than 6.4. The curd was cut into about 1.5 to 2.0 cm^3^ cubes and scalded to 45 °C (rising by 1 °C every 3 min). Then, draining, salting (0.9 g 100 g^−1^ sodium chloride), molding, and pressing in a manual press were performed. During pressing, each cheese was turned three times (each turn lasted 1 h), and the total pressing time was 4 h. The resulting cheese yield was measured by the ratio of liters of milk used to produce one kilogram of cheese (L/kg) [25].

The F cheeses were vacuum-packed the next day after manufacture. During the ripening stage, the cheeses were unmolded and placed in plastic trays, then stored in a cold chamber at 5 °C with a relative humidity of 75%. The cheeses were vacuum-packed after completing the ripening phase. The cheese production steps followed the norms of Regulation No. 146 [26].

### 2.4. Chemical Composition of Cheese Samples

The chemical composition of cheese samples was determined in triplicate according to the official methods of AOAC International [27], analyzing pH, total titratable acidity, moisture, and total solids and ash. Water activity was measured on an a_w_ meter (LabSwift-Novasina, Piracicaba-SP, Brazil). A conversion factor of 6.38 was used to determine proteins after total nitrogen analysis following the Kjeldahl method (AOAC, 1970). The fat content was determined by the Gerber methodology, described in Regulation No. 68 [26].

### 2.5. Colorimetric Analysis

The color of cheese samples was analyzed by an instrumental method using an ACR-1023^®^ colorimeter (Instrutherm, São Paulo, Brazil) in the RGB system and converted into CIELAB by the OpenRGB^®^ program (Logicol, Italy). The L* coordinate readings were taken to assess the luminosity (L* = 0, black; L* = 100, white), while a* and b* refer to the chromaticity coordinates: a* represents the variation degree between green and red (a* negative = green; a* positive = red), and b* expresses a hue between blue and yellow (b* negative = blue; b* positive = yellow) [28]. The device was previously calibrated, and the colorimetry readings were performed in the geometric center of each cheese in triplicate. The Yellowness Index (YI) was calculated from the average of the values of L * a * b *, according to the following equation [29]:

YI = 142.86 (b*/L*)

### 2.6. Texture Profile Analysis

The Texture Profile Analysis (TPA) was performed in a universal texturometer (TA-XT plus, Stable MicroSystems, Godalming, UK) equipped with integrated Exponent Stable MicroSystems 32 software, 36 mm AACC cylindrical probe (model P-36R), and a 30 kg load cell. The double compression method was used, simulating chewing, with two cycles (20% compression; 5 s between cycles; constant speed of 2 mm/s). The cheese samples were 20 mm in diameter and were kept at room temperature (30 °C) for around 4 h before testing [30]. The firmness, cohesiveness, chewability, and resilience parameters were analyzed. A total of four repetitions were performed for each cheese sample.

### 2.7. Microbiological Analyses

The different formulations (milk: colostrum) used to produce the cheeses were subjected to the analysis of total and thermotolerant coliforms (MPN/g) after the pasteurization process to verify their hygienic quality [31].

All the different formulations of fresh cheeses were subjected to the analysis of thermotolerant coliforms (MPN/g), molds and yeasts, coagulase-positive staphylococci (*Staphylococcus aureus*), *Listeria monocytogenes*, and *Salmonella* spp. [32]. The results were evaluated based on the specific quality parameters for cheeses established by the Brazilian health agency Agência Nacional de Vigilância Sanitária [26,32,33,34].

### 2.8. Sensory Analysis

Hedonic sensory tests were carried out by a panel of 93 untrained volunteer tasters (ranging from 18 to 51 years), recruited in November 2019, among students and workers from the Federal University of Rio Grande do Norte (Escola Agrícola de Jundiaí, Macaíba, RN, Brazil).

For conducting sensory tests, this work was approved by the Research Ethics Committee of the Health Sciences College of Trairi, Universidade Federal of Rio Grande do Norte (UFRN), under approval number 3.696.904. The written informed consent was properly collected. The authors had no access to information that could identify individual participants during or after data collection.

The tests occurred in individual sensory analysis booths, with temperature controlled to 22 °C and artificial white light. Water and salty crackers were offered to the volunteers between the samples. The evaluated parameters were appearance, color, aroma, texture, flavor, and overall acceptance using a hedonic scale of 9 points, ranging from disliked very much (1) to liked very much (9). Cheeses were also evaluated for purchase intent by a numerical and nominal five-point scale (1 = certainly would not buy, to 5 = would certainly buy).

The Acceptability Index (AI) was calculated according to Dutcosky [35], which classifies a product with good acceptability when the AI is greater than 70, using the equation:

AI (%) = M/h × 100

in which:

M = arithmetic mean of the scores assigned to the parameter, and

h = highest score given by the tasters to the parameter under analysis.

### 2.9. Statistical Analyses

The results were expressed as means and standard deviations. Differences between treatments were determined through an analysis of variance (ANOVA) with the R version 3.5.0 software program using the “Agricolae” package, complemented by the Tukey test with a 0.05-significance level. The results of the acceptability tests for sensory attributes and purchase intention were subjected to the Dunnet test by comparing cheeses with the colostrum addition to the control sample.

## 3. Results and Discussion

The characteristics of the milk and colostrum used to manufacture the cheeses can be seen in Table 1. The milk composition is within the legal standards of Regulation No. 76 (IN 76) of the Ministry of Agriculture, Livestock and Supply [28].

The average colostrum composition values are higher when compared with milk, except for lactose and fat, which had a lower concentration. Conte and Scarantino [36] report that a gradual increase in lactose values during lactation is expected, particularly in the first days after calving. Sobczuk-Szul et al. [37] show 23.34 g 100 g^−1^ for solids-not-fat in colostrum from Jersey cows, which is higher than the value found in this study (17.46 g 100 g^−1^). The 23 g 100 g^−1^ total solids value colostrum from Jersey dairy cows is reported by Morrill et al. [38], being slightly higher than that found in the present study (21.52 g 100 g^−1^).

Oliveira et al. [39] report that the high protein concentration in colostrum is related to the greater amount of casein and immunoglobulins. These immunoglobulins have the function of protecting the calf from various diseases until its body is able to develop its own defense cells. Clinical trials have proven that bovine colostrum immunoglobulins may also benefit human health. Among them, Potiroglu and Kondolot [40] conducted a study on using bovine colostrum in treating children with upper respiratory tract infections due to IgA deficiency. They observed a reduction in the severity of the infections. Those researchers emphasize that no adverse effects are followed by the patients’ mothers. The use of colostrum for producing immunoglobulins on an industrial scale is interesting because it has high bioavailability and safety compared to blood products [23]. This makes bovine colostrum a source of bioactive compounds of interest for developing functional foods.

Colostrum shows a pH of 6.4 and acidity of 0.25 g lactic acid/100 mL (Table 1). The colostrum pH is higher than that reported by Saalfeld et al. [41] with a pH of 6.29, while the acidity value is similar to colostrum within 24 h from calving (0.25 g of lactic acid/100 mL; [41]). The acidity value is probably related to the presence of solids-not-fat, such as albumin, caseins, and phosphates; thus, when colostrum is characterized by high protein content, high acidity is expected [41]. Nardone et al. [42] also observe higher titratable acidity for colostrum when compared to milk and report a positive correlation with its protein content.

Regarding the composition of cheeses made by processing milk with colostrum (Table 2), moisture results ranged from 44.77 to 63.22 g 100 g^−1^. Differences in moisture between the samples according to the colostrum level and the maturation time (*p* < 0.05) are observed. All the fresh cheese formulations with added colostrum (1F, 2F, and 3F) can be classified as very high moisture cheeses, as their moisture content is greater than 55.0 g 100 g^−1^. The 0B and 1C cheeses are within the standards for medium-moisture cheeses, with percentages within 44.77 to 45.9 g 100 g^−1^ [23]. The remaining aged cheeses presented moisture ranging from 46.59 to 63.22 g 100 g^−1^; therefore, they were classified as high-moisture (46–54.9 g 100 g^−1^) to very high-moisture cheeses (above 55 g 100 g^−1^) according to the Brazilian regulation [26]. As expected, the moisture content of the cheese samples is directly linked to the maturation time applied. Cheeses with a short maturation time tend to be moister than cheeses with a lengthier maturation time [43]. Evaluating the characteristics of rennet-type cheese produced using cow’s milk, Roig et al. [44] and Santos et al. [45] observed that the high moisture of the cheeses was due to the greater presence of denatured whey proteins because of the use of pasteurized milk. When analyzing the variability of cheese moisture content in this study, the pasteurization effect seems to affect cheese obtained from milk added with colostrum. According to Souza and Saad [46] and Santos et al. [45], another explanation for the difference in cheese moisture would be related to milk and colostrum pH, as the concentration of hydrogen ions leads to a reduction in repulsive forces and a consequent increase in the aggregation of casein micelles, which may explain the lower moisture retention in cheese obtained from only milk.

The treatments with the addition of 15 and 25 mL of colostrum per 100 mL of milk in cheeses matured for 20 and 40 days (3B and 1C, respectively) yielded the highest ash values. The simultaneous effect of colostrum addition and maturation promotes a higher concentration of this component in cheese. Indeed, significant differences (*p* < 0.05) are found between different concentrations of colostrum additions, as well as between maturation times (*p* < 0.05). Our cheeses added with colostrum reached a higher ash content than those reported by Assunção et al. (3.02 to 3.24 g 100 g^−1^) for similar types of artisanal spicy cheeses without colostrum [47].

Among the treatments, type 0B cheese is classified as fatty cheese, as it has fat in the dry matter within the limits of 45.0 to 59.9 g 100 g^−1^ [26]. Cheeses 0B and 0F showed the highest percentages of FDM with significant differences (*p* < 0.05) compared to the other formulations. The 2A cheese sample has the lowest lipid content which is consequently reflected in the FDM (21.21 g 100 g^−1^), constituting the lowest value among all treatments. Thus, according to Brazilian standards, cheese 2A is classified as reduced fat (fat content between 10.0 and 24.9 g 100 g^−1^) [26]. All the other cheeses are classified as within the semi-fat criteria, including those with fat content in the dry matter between 25.0 and 44.9 g 100 g^−1^ [26]. The reduction in the lipid index observed in the cheeses with added colostrum can be explained by the differences between the high lipid content found in the fresh milk used for making the cheeses (5.4 g 100 g^−1^) and the percentage of this component in the 24-h colostrum (4.05 g 100 g^−1^). It may also be due to the formation of more fragile curds, which, according to Sousa et al. [48], affect the ability to retain fat, influencing the centesimal composition.

Cheeses produced from a different proportion of milk and colostrum are also significantly different regarding SNF (*p* < 0.05). This trait is important because it allows the assessment of fat expressed in relation to total dry matter (TDM), correcting for the variation that may occur because of moisture losses [49]. The control cheese (0B) shows the highest FDM, with this being justified by the higher fat content (5.40 g 100 g^−1^) of milk compared to that in the colostrum (4.05 g 100 g^−1^). According to Foley and Otterby [2], bovine colostrum fat decreases with the time interval from calving (fat equal to 6.7 g 100 g^−1^ postpartum, 5.4 g 100 g^−1^ at 24 h, and 3.9 g 100 g^−1^ at 72 h). Raimondo et al. [50] reported a high quantity of fat colostrum in Jersey cows, from 1.35 ± 1.17 g/dL on the first day from calving to 3.09 ± 2.19 g/dL on the third day of lactation.

The protein content of cheese is similar among treatments, although the percentage was slightly lower in samples without colostrum addition. This can be justified by the higher whey protein content in colostrum and the low proportion of milk’s total protein content [51].

The titratable acidity of cheese (Table 2) is expressed as a percentage of lactic acid and varied between 0.02 to 0.08 among treatments, presenting a lower variability compared to that reported by Sousa et al. [48] for traditional rennet cheese (0.12 to 1.01). Those authors stated that the acidity resulting from lactic acid production has a direct influence on the expulsion of whey, especially during syneresis. In addition, acidity can influence the texture [52], microbial activity, and maturation index of cheese [48]. Thus, the low titratable acidity observed in this study reflects the pH values found in the cheese formulations.

Cheese pH mean values (Table 2) range from 5.02 (3C) to 6.23 (3F). The pH of the fresh cheeses among treatments gradually increased as the colostrum proportion in the formulation increased. A similar pH was found by Simon and coworkers for a cheese made with 100% colostrum (6, 15) [18]. The presence of immunoglobulins, considered the main antimicrobial factor of colostrum [50], possibly affected the fermentation process by the lactic acid bacteria added to the cheeses, even if the proteins that remain soluble (including immunoglobulins) are drained in the whey during curd formation. Further studies are needed to analyze the lactic acid bacteria action in cheese fermentation using milk added with colostrum.

Water activity values of cheese samples vary between 0.94 and 0.99 (*p* < 0.05; Table 2). It is known that cheeses with high water activity levels are more susceptible to high microbial development [53]. Thus, the activity is inversely proportional to the shelf life of cheeses. In general, the longer the ripening period, the lower the water activity; however, in our experiments, no significant differences were observed between ripe and fresh cheeses, meaning the maturation time was not long enough to produce changes in a_w_.

Regarding the measurement of color, we report that the variation in the colostrum added to milk produces differences among cheese samples (Table 3). The cheeses of the present study generally show medium luminosity (L*), with a predominance of yellow (b*) and green (a*). Thus, the cheeses analyzed herein were characterized as yellowish-white, resembling the characteristics of rennet cheese. The L* brightness values ranged from 55.43 to 83.44. The negative a* value represents the intensity of the green color, while the positive value refers to the red one. The b* values ranged from 14.07 to 34.04, representing the intensity of the yellow color.

Generally, increased yellowing is directly related to the amount of fat due to the saturation of adipocytes with beta-carotene from animal metabolism [54,55]. During ripening, the concentration of non-aqueous components usually leads to color changes. Although slight differences in the yellowing index (YI) between fresh and matured cheeses can be observed in this study (40-day ripe cheeses achieved YI higher than the fresh formulations), there was no statistical relevance of maturation time on this parameter, except for the cheese produced from milk with the addition of 20 mL 100 mL^−1^ colostrum.

Table 4 presents the characteristics of the cheese texture profile among treatments. According to Fontan [56], cheese texture is affected by composition and maturation. The F0 and A0 samples showed greater firmness (*p* < 0.05). There is a tendency to lower firmness in cheeses with increasing colostrum levels in the formulation. These data are consistent with cheese moisture since they present a greater solids dispersion and, therefore, a lower firmness.

Pereira et al. [57] studied the correlation between the instrumental and sensory texture of several similar commercial types of cheeses. They found that cheeses with low moisture content were generally the firmest. Such findings confirm the results of this work since the control cheese with the lowest moisture percentage (47.52 g 100 g^−1^) was the firmest, while the cheeses with the colostrum addition had the highest moisture content and the lowest firmness.

Cohesiveness is instrumentally assessed as the amount of energy necessary to break the internal structure of the cheese, and in sensory analysis, it is defined as the degree to which cheese is compressed between the teeth before breaking [58]. In the present study, this parameter ranged from 0.34 to 0.57, with fresh control cheese having the lowest value (*p* < 0.05). Some of the values found in this study are close to the work of Andrade et al. [59] in evaluating industrial and artisanal cheeses, finding results ranging from 0.59 to 0.67 and 0.49 to 0.65, respectively.

Among all the treatments, 0F and 0A showed greater chewability (*p* < 0.05). According to the results obtained for this parameter, it is possible to observe that cheeses produced from formulations with higher amounts of colostrum were characterized by lower firmness. This phenomenon may possibly be associated with higher moisture content, as water in cheese acts together with fat as a lubricant between casein aggregates [58]. Thus, the reduction in moisture content results in an increase in cheese hardness and chewability.

**Figure 1:**
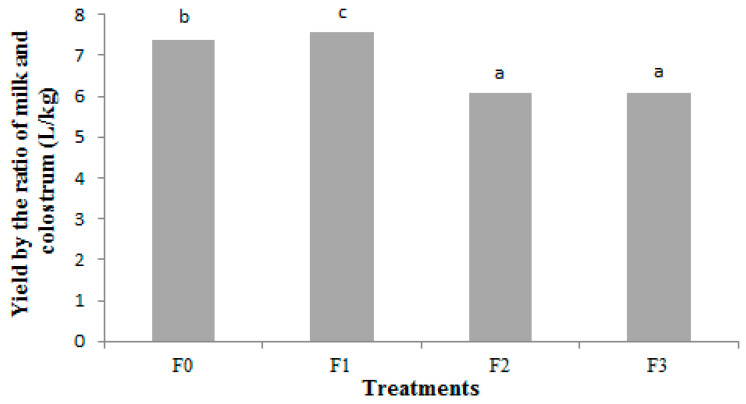
Average of fresh (F) cheese yield (L/kg) from cheese formulations added with different levels of bovine colostrum 0:100, 15:85, 20:80, and 25:75 mL 100 mL^−1^, bovine colostrum:milk (*v*:*v*)—F0, F1, F2, and F3, respectively. Means identified with different letters differ from each other by the Tukey test (*p* < 0.05).

Cheese yield is primarily due to the milk quantity and the recovery in the curd of the protein and fat from the milk to the cheese [53]. Figure 1 shows the cheese yield calculated in liters of milk per kilogram of cheese (L/kg), with the best performance for the formulation with the higher percentage of colostrum (6.08 L needed to produce one kg of cheese). There is also a significant difference (*p* < 0.05) between the formulations without colostrum and with the lowest percentage of colostrum (15 mL).

All the cheeses in this experiment were produced with similar production technology to rennet cheese processing and showed a ratio of the amount of milk and colostrum below 10 L of the total volume for each kilogram of cheese produced, which indicates a good yield when compared to cheeses with the same moisture content [60,61]. According to Dutra [62], the average yield for rennet cheese manufacturing by the traditional process is between 10.5 and 12.5 L of milk per kilogram of cheese.

The microbiological profile of milk, colostrum, and cheese samples met the Brazilian criteria determined by the national regulatory agency [26,33,34], which establishes the absence of *Salmonella* spp. and *Listeria monocytogenes* in a 25 g sample, and a tolerance limit of 1 × 10^3^ MPN/g for coagulase-positive staphylococci, 1 × 10^4^ MPN/g for total coliforms, 5 × 10^3^ MPN/g for thermotolerant coliforms, and 5.0 × 10^3^ UFC/g for molds and yeasts.

Table 5 presents the descriptive statistics of sensory attributes obtained for the fresh cheese among different treatments. The appearance and color parameters scores ranged from 7.67 to 8.10 and 7.54 to 7.81, respectively, with no statistical differences (*p* < 0.05) in these attributes between the samples.

The control cheese had the highest average for aroma (7.52), texture (7.83), and flavor (7.90) compared to the other treatments (*p* < 0.05). The control cheese also obtained a significantly higher overall evaluation (7.72). It is worth noting that despite the statistical differences, cheese from milk added with 25 mL 100 mL^−1^ of colostrum is the treatment showing the closest scores to the control one in terms of overall evaluation and purchase intent.

Moreover, the control cheese obtained the highest AI for all evaluated attributes (Table 6). However, the 3F treatment scored the highest AI among cheeses produced from milk added with colostrum, except for the appearance and color attributes for which the 2F obtained the highest value.

It was further observed that all the evaluated cheese formulations obtained a satisfactory AI. Dutcosky [35] suggested that a food product with good acceptability presents average AI values above 70. Good acceptance of colostrum-added dairy products has previously been reported; Saalfeld et al. [41] observed sensory approval of dairy beverages enriched with colostrum silage. Mouton and Aryana [19] analyzed the influence of colostrum on the characteristics of ice cream and recommended such an industrial application based on a sensory analysis by trained panelists.

## 4. Conclusions

Part of the colostrum cows produce is a surplus dairy farm product. However, there are challenges to appropriately designate the daily surplus of colostrum production and a need for processing and product preservation technologies. Based on the overall assessment and the purchase intention of the fresh cheeses analyzed in this study, it is possible to speculate that all cheeses made using formulations with colostrum have the potential to be used for human consumption. Among the cheeses with colostrum, the fresh cheese with 75:25 milk:colostrum (*v*:*v*) received the highest scores for the aroma, texture, flavor, overall evaluation, and purchase intention parameters. Furthermore, this formulation (25% colostrum) achieved a better yield than those without or with 15% colostrum. Regarding texture measurements, after 40 days of maturation, the proportion of 25% colostrum reached cohesivity, resilience, and bright color significantly higher than its fresh correspondent.

This study contributes to knowledge on the use of colostrum in elaborating and enriching dairy derivatives intended for human consumption, as the use of colostrum is little known culturally in Brazil and many other countries. In addition, the results herein point to a product with innovative appeal, strengthening research in seeking technologies and innovations to use this raw material, which is still underutilized and wasted due to excess production.

## Figures and Tables

**Table 1:** Composition, pH, and acidity of milk and colostrum used in cheese production (mean ± standard deviation).

Composition	Milk ± SD	Colostrum ± SD
Fat (g 100 g^−1^)	5.40 ± 0.01 ^a^	4.05 ± 0.01 ^b^
Total protein (g 100 g^−1^)	3.87 ± 0.01 ^b^	15.20 ± 0.01 ^a^
Lactose (g 100 g^−1^)	5.34 ± 0.01 ^a^	1.48 ± 0.01 ^b^
Casein (g 100 g^−1^)	3.02 ± 0.01 ^b^	12.07 ± 0.01 ^a^
SNF (g 100 g^−1^)	10.02 ± 0.01 ^b^	17.46 ± 0.14 ^a^
TS (g 100 g^−1^)	15.42 ± 0.01 ^a^	21.52 ± 0.01 ^b^
Acidity (g lactic acid 100 mL^−1^)	0.18 ± 0.01 ^b^	0.25 ± 0.01 ^a^
pH	6.80 ± 0.01 ^a^	6.40 ± 0.01 ^b^

SNF: Solids-not-fat; TS: Total solids. SD: Standard Deviation. Means with different letters on the same line differ from each other by the Tukey test (*p* < 0.05).

**Table 2:** Composition of cheeses enriched with different levels of bovine colostrum and matured for 10, 20, and 40 days (mean ± standard deviation).

Treatment	Moisture	Ash	FDM	Protein	Acidity	pH	a_w_
0—100:0 (milk:colostrum, *v*:*v*)							
F	47.52 ± 0.16 ^g^	2.95 ± 0.00 ^f^	41.09 ± 5.52 ^ab^	22.24 ± 0.21 ^c^	0.04 ± 0.00 ^b^	5.39 ± 0.02 ^ef^	0.95 ± 0.01 ^b^
A	46.59 ± 0.29 ^h^	3.53 ± 0.05 ^cde^	31.00 ± 1.91 ^def^	25.48 ± 0.57 ^abc^	0.04 ± 0.01 ^b^	5.65 ± 0.08 ^bc^	0.95 ± 0.01 ^ab^
B	44.77± 0.11 ^i^	3.34 ± 0.06 ^ef^	47.36 ± 0.98 ^a^	23.06 ± 1.32 ^bc^	0.05 ± 0.00 ^ab^	5.50 ± 0.02 ^de^	0.99 ± 0.01 ^a^
C	49.11± 0.17 ^f^	3.96 ± 0.04 ^bc^	32.60 ± 0.19 ^cde^	25.85 ± 0.49 ^ab^	0.08 ± 0.01 ^a^	5.22 ± 0.02 ^gh^	0.97 ± 0.00 ^ab^
1—85:15 (milk:colostrum, *v*:*v*)							
F	56.98 ± 0.22 ^b^	3.44 ± 0.01 ^de^	26.24 ± 1.33 ^efg^	25.30 ± 0.16 ^abc^	0.03 ± 0.01 ^b^	5.61 ± 0.09 ^bc^	0.96 ± 0.01 ^b^
A	51.00 ± 0.13 ^e^	3.32 ± 0.08 ^ef^	32.58 ± 0.08 ^cde^	23.45 ± 017 ^abc^	0.02 ± 0.00 ^b^	5.24 ± 0.03 ^gh^	0.95 ± 0.02 ^ab^
B	52.41 ± 0.41 ^d^	3.63 ± 0.01 ^bcde^	38.40 ± 0.96 ^bc^	26.07 ± 2.40 ^ab^	0.03 ± 0.01 ^b^	5.68 ± 0.00 ^bc^	0.96 ± 0.01 ^ab^
C	45.99 ± 0.19 ^h^	4.92 ± 0.02 ^a^	26.87 ± 1.32 ^efg^	24.00 ± 0.49 ^abc^	0.08 ± 0.01 ^a^	5.30 ± 0.02 ^fg^	0.94 ± 0.01 ^b^
2—80:20 (milk:colostrum, *v*:*v*)							
F	63.22 ± 0.16 ^a^	3.21 ± 0.02 ^ef^	31.56 ± 1.52 ^def^	24.66 ± 0.25 ^abc^	0.04 ± 0.00 ^b^	5.73 ± 0.01 ^b^	0.97 ± 0.01 ^ab^
A	54.58 ± 0.11 ^c^	4.01 ± 0.09 ^b^	21.21 ± 0.65 ^g^	26.65 ± 0.33 ^a^	0.04 ± 0.02 ^ab^	5.57 ± 0.03 ^cd^	0.96 ± 0.00 ^ab^
B	47.48 ± 0.37 ^g^	3.85 ± 0.03 ^bcd^	35.65 ± 0.34 ^bcd^	25.58 ± 0.05 ^abc^	0.02 ± 0.00 ^b^	5.48 ± 0.02 ^de^	0.97 ± 0.02 ^ab^
C	49.42 ± 0.11 ^f^	3.84 ± 0.09 ^bcd^	35.13 ± 0.29 ^bcd^	25.42 ± 0.03 ^abc^	0.03 ± 0.01 ^b^	5.03 ± 0.01 ^ij^	0.95 ± 0.01 ^ab^
3—75:25 (milk:colostrum, *v*:*v*)							
F	57.13 ± 0.69 ^b^	3.23± 0.12 ^ef^	40.29 ± 3.89 ^b^	24.73 ± 1.51 ^abc^	0.02 ± 0.00 ^b^	6.23 ± 0.01 ^a^	0.95 ± 0.03 ^b^
A	53.06± 0.12 ^d^	3.45 ± 0.02 ^de^	32.68 ± 3.18 ^cde^	23.83 ± 0.63 ^abc^	0.04 ± 0.01 ^b^	5.14 ± 0.01 ^hi^	0.95 ± 0.01 ^ab^
B	51.49 ± 0.14 ^e^	4.90 ± 0.55 ^a^	25.42 ± 3.16 ^fg^	23.22 ± 0.25 ^bc^	0.02 ± 0.00 ^b^	5.46 ± 0.00 ^de^	0.96 ± 0.00 ^ab^
C	51.31 ± 0.27 ^e^	3.53 ± 0.01 ^cde^	29.65 ± 1.18 ^def^	25.00 ± 0.44 ^abc^	0.03 ± 0.01 ^b^	5.02 ± 0.01 ^j^	0.95 ± 0.01 ^b^

FDM: Fat in Dry Matter; Moisture, ash, crude protein, and acidity are expressed in g 100 g^−1^; 0: control samples (0 colostrum addition); 1: Cheese produced from milk with the addition of 15 mL 100 mL^−1^ colostrum; 2: Cheese produced from milk with the addition of 20 mL 100 mL^−1^ colostrum; 3: Cheese produced from milk with the addition of 25 mL 100 mL^−1^ colostrum; F: fresh cheese; A: Cheese matured for 10 days; B: Cheese matured for 20 days; C: Cheese matured for 40 days. Means with different letters in the same column differ from each other by the Tukey test (*p* < 0.05).

**Table 3:** Color characteristics of cheeses enriched with different levels of bovine colostrum and matured for 10, 20, and 40 days (mean ± standard deviation).

Treatments	L*	a*	b*	YI
0—100:0 (milk:colostrum, *v*:*v*)				
F	65.80 ± 7.87 ^def^	−6.83 ± 8.78 ^abc^	17.04 ± 5.68 ^d^	37.24 ± 12.81 ^bc^
A	62.38 ± 1.89 ^efg^	−5.57 ± 5.13 ^abc^	19.01 ± 4.50 ^bcd^	43.62 ± 10.71 ^abc^
B	64.65 ± 3.13 ^def^	7.21 ± 8.62 ^a^	17.83 ± 12.65 ^cd^	39.35 ± 27.94 ^bc^
C	75.66 ± 0.66 ^b^	−7.76 ± 1.91 ^abc^	30.73 ± 0.65 ^abc^	58.01 ± 0.72 ^abc^
1—85:15 (milk:colostrum, *v*:*v*)				
F	62.76 ± 0.86 ^defg^	1.49 ± 5.22 ^ab^	20.09 ± 1.88 ^bcd^	45.69 ± 3.63 ^abc^
A	59.77 ± 0.86 ^fg^	−7.53 ± 1.36 ^abc^	23.69 ± 0.27 ^abcd^	56.62 ± 0.34 ^abc^
B	63.26 ± 3.30 ^def^	−10.28 ± 3.60 ^bc^	15.30 ± 1.54 ^d^	34.51 ± 1.88 ^c^
C	64.48 ± 0.40 ^def^	2.16 ± 1.49 ^ab^	31.49 ± 1.80 ^ab^	69.78 ± 4.40 ^a^
2—80:20 (milk:colostrum, *v*:*v*)				
F	63.53 ± 1.84 ^def^	−15.06 ± 6.09 ^c^	16.70 ± 3.26 ^d^	37.54 ± 7.03 ^bc^
A	83.44 ± 1.03 ^a^	−5.39 ± 4.22 ^abc^	26.99 ± 3.92 ^abcd^	46.16 ± 6.18 ^abc^
B	66.51 ± 2.52 ^cdef^	−8.60 ± 0.92 ^abc^	25.25 ± 4.38 ^abcd^	54.06 ± 7.47 ^abc^
C	68.79 ± 1.07 ^bcde^	7.04 ± 0.79 ^a^	34.04 ± 2.71 ^a^	70.64 ± 4.64 ^a^
3—75:25 (milk:colostrum, *v*:*v*)				
F	55.43 ± 0.87 ^g^	−4.10 ± 6.24 ^abc^	14.07 ± 3.81 ^d^	36.19 ± 9.30 ^c^
A	74.16 ± 0.51 ^bc^	0.02 ± 4.00 ^abc^	33.66 ± 0.01 ^a^	64.84 ± 0.44 ^ab^
B	64.68 ± 1.88 ^def^	3.69 ± 6.07 ^ab^	24.82 ± 1.04 ^abcd^	54.79 ± 0.92 ^abc^
C	70.41 ± 0.51 ^bcd^	1.89 ± 9.43 ^ab^	26.06 ± 1.29 ^abcd^	52.88 ± 2.65 ^abc^

0: control samples (0 colostrum addition); 1: Cheese produced from milk with the addition of 15 mL 100 mL^−1^ colostrum; 2: Cheese produced from milk with the addition of 20 mL 100 mL^−1^ colostrum; 3: Cheese produced from milk with the addition of 25 mL 100 mL^−1^ colostrum; F: fresh cheese; A: Cheese matured for 10 days; B: Cheese matured for 20 days; C: Cheese matured for 40 days. Means with different letters in the same column differ from each other by the Tukey test (*p* < 0.05).

**Table 4:** Texture characteristics of cheeses enriched with different levels of bovine colostrum and matured for 10, 20, and 40 days (mean ± standard deviation).

Treatments	Firmness	Cohesivity	Chewability	Resilience
0—100:0 (milk:colostrum, *v*:*v*)				
F	58.99 ± 6.51 ^ab^	0.60 ± 0.05 ^a^	35.89 ± 6.96 ^ab^	0.28 ± 0.03 ^b^
A	65.20 ± 8.64 ^a^	0.61 ± 0.05 ^a^	40.09 ± 6.56 ^a^	0.29 ± 0.04 ^b^
B	50.03 ± 8.08 ^bc^	0.55 ± 0.03 ^ab^	27.41 ± 5.00 ^bcd^	0.22 ± 0.03 ^b^
C	46.31 ± 4.53 ^bcd^	0.61 ± 0.05 ^a^	28.14 ± 3.69 ^bc^	0.20 ± 0.02 ^b^
1—85:15 (milk:colostrum, *v*:*v*)				
F	33.44 ± 3.25 ^def^	0.56 ± 0.03 ^ab^	18.62 ± 1.15 ^def^	0.31 ± 0.06 ^b^
A	42.87 ± 4.82 ^cd^	0.53 ± 0.05 ^ab^	22.76 ± 3.82 ^cde^	0.32 ± 0.08 ^b^
B	26.37 ± 1.20 ^efg^	0.50 ± 0.02 ^abc^	13.22 ± 0.81 ^fg^	0.44 ± 0.27 ^b^
C	39.21 ± 6.91 ^cde^	0.34 ± 0.06 ^d^	13.39 ± 4.37 ^fg^	0.39 ± 0.13 ^b^
2—80:20 (milk:colostrum, *v*:*v*)				
F	11.75 ± 1.26 ^h^	0.40 ± 0.08 ^cd^	4.68 ± 0.80 ^g^	0.39 ± 0.18 ^b^
A	51.07 ± 4.5 ^bc^	0.51 ± 0.04 ^abc^	25.68 ± 3.72 ^cd^	0.45 ± 0.18 ^b^
B	45.34 ± 7.08 ^cd^	0.57 ± 0.04 ^ab^	25.64 ± 2.79 ^cd^	0.22 ± 0.02 ^b^
C	27.58 ± 2.26 ^efg^	0.45 ± 0.03 ^bcd^	12.56 ± 1.57 ^fg^	0.40 ± 0.53 ^b^
3—75:25 (milk:colostrum, *v*:*v*)				
F	16.46 ± 1.86 ^gh^	0.55 ± 0.03 ^ab^	8.99 ± 1.03 ^g^	0.23 ± 0.04 ^b^
A	23.51 ± 1.69 ^fgh^	0.55± 0.06 ^ab^	12.93 ± 2.22 ^fg^	0.25 ± 0.11 ^b^
B	24.24 ± 4.96 ^fgh^	0.57 ± 0.04 ^ab^	13.84 ± 3.53 ^efg^	0.22 ± 0.03 ^b^
C	23.34 ± 3.24 ^fgh^	0.41 ± 0.05 ^cd^	9.46 ± 0.69 ^fg^	1.14 ± 0.23 ^a^

0: control samples (0 colostrum addition); 1: Cheese produced from milk with the addition of 15 mL 100 mL^−1^ colostrum; 2: Cheese produced from milk with the addition of 20 mL 100 mL^−1^ colostrum; 3: Cheese produced from milk with the addition of 25 mL 100 mL^−1^ colostrum; F: fresh cheese; A: Cheese matured for 10 days; B: Cheese matured for 20 days; C: Cheese matured for 40 days. Means with different letters in the same column differ from each other by the Tukey test (*p* < 0.05).

**Table 5:** Scores attributed to appearance, color, aroma, texture, taste, overall evaluation (9-point hedonic scale), and purchase intention (5-point scale) for fresh cheeses with added bovine colostrum at the levels of 0:100, 15:85, 20:80, and 25:75, bovine colostrum:milk (*v*:*v*) (mean ± standard deviation).

Attributes	0F	1F	2F	3F
Appearance	8.10 ± 0.99 ^a^	7.67 ± 1.34 ^a^	7.86 ± 1.09 ^a^	7.75 ± 1.22 ^a^
Color	7.81 ± 1.31 ^a^	7.54 ± 1.26 ^a^	7.73 ± 1.17 ^a^	7.64 ± 1.38 ^a^
Aroma	7.52 ± 1.40 ^a^	6.76 ± 1.63 ^b^	7.07 ± 1.46 ^b^	7.12 ± 1.62 ^b^
Texture	7.83 ± 1.16 ^a^	6.98 ± 1.97 ^b^	6.98 ± 1.74 ^b^	7.36 ± 1.61 ^b^
Flavor	7.90 ± 1.32 ^a^	6.63 ± 1.96 ^b^	6.47 ± 1.93 ^b^	7.07 ± 1.80 ^b^
Overall evaluation	7.72 ± 1.02 ^a^	7.08 ± 1.52 ^b^	6.94 ± 1.56 ^b^	7.41 ± 1.40 ^b^
Purchase intent	4.38 ± 0.94 ^a^	3.49 ± 1.16 ^b^	3.50 ± 1.17 ^b^	3.79 ± 1.28 ^b^

0: control sample (0 colostrum addition); 1: Cheese produced from milk with the addition of 15 mL 100 mL^−1^ colostrum; 2: Cheese produced from milk with the addition of 20 mL 100 mL^−1^ colostrum; 3: Cheese produced from milk with the addition of 25 mL 100 mL^−1^ colostrum; F: fresh cheese. Means with different letters on the same line differ from each other by the Dunnet test (*p* < 0.05).

**Table 6:** Acceptability Index (IA) of fresh cheeses obtained from milk with different colostrum additions.

Attributes	AI (%)			
	0F	1F	2F	3F
Appearance	90.08	85.30	87.33	86.14
Color	86.85	83.87	85.90	84.94
Aroma	83.63	75.14	78.49	79.21
Texture	87.09	75.12	77.65	81.83
Flavor	87.81	73.71	71.92	78.61
Overall evaluation	86.97	78.73	77.18	82.43

AI: Acceptability Index; 0F: fresh cheese with 0 colostrum; 1F, 2F and 3F: Fresh cheeses with the additions of 15, 20, and 25 mL 100 mL^−1^ colostrum, respectively.

## Data Availability

The data used to support the findings of this study can be made available by the corresponding author upon request.
